# Wilson’s Disease—Crossroads of Genetics, Inflammation and Immunity/Autoimmunity: Clinical and Molecular Issues

**DOI:** 10.3390/ijms25169034

**Published:** 2024-08-20

**Authors:** Grażyna Gromadzka, Julia Czerwińska, Elżbieta Krzemińska, Adam Przybyłkowski, Tomasz Litwin

**Affiliations:** 1 Department of Biomedical Sciences, Faculty of Medicine, Collegium Medicum, Cardinal Stefan Wyszynski University, Wóycickiego Street 1/3, 01-938 Warsaw, Poland; 2 Students Scientific Association “Immunis”, Cardinal Stefan Wyszynski University, Dewajtis Street 5, 01-815 Warsaw, Poland; 3 Department of Gastroenterology and Internal Medicine, Medical University of Warsaw, Banacha 1a, 02-097 Warsaw, Poland; aprzybylkowski@wum.edu.pl; 4 Second Department of Neurology, Institute of Psychiatry and Neurology, Sobieskiego Street 9, 02-957 Warsaw, Poland; tomlit@medprakt.pl

**Keywords:** Wilson’s disease, *ATP7B*, immunology, copper, cuproptosis, ferroptosis, oxidative stress, autoimmunity, autoantibodies, inflammation

## Abstract

Wilson’s disease (WD) is a rare, autosomal recessive disorder of copper metabolism caused by pathogenic mutations in the *ATP7B* gene. Cellular copper overload is associated with impaired iron metabolism. Oxidative stress, cuproptosis, and ferroptosis are involved in cell death in WD. The clinical picture of WD is variable. Hepatic/neuropsychiatric/other symptoms may manifest in childhood/adulthood and even old age. It has been shown that phenotypic variability may be determined by the type of *ATP7B* genetic variants as well as the influence of various genetic/epigenetic, environmental, and lifestyle modifiers. In 1976, immunological abnormalities were first described in patients with WD. These included an increase in IgG and IgM levels and a decrease in the percentage of T lymphocytes, as well as a weakening of their bactericidal effect. Over the following years, it was shown that there is a bidirectional relationship between copper and inflammation. Changes in serum cytokine concentrations and the relationship between cytokine gene variants and the clinical course of the disease have been described in WD patients, as well as in animal models of this disease. Data have also been published on the occurrence of antinuclear antibodies (ANAs), antineutrophil cytoplasmic antibodies (ANCAs), anti-muscle-specific tyrosine kinase antibodies, and anti-acetylcholine receptor antibodies, as well as various autoimmune diseases, including systemic lupus erythematosus (SLE), myasthenic syndrome, ulcerative colitis, multiple sclerosis (MS), polyarthritis, and psoriasis after treatment with d-penicillamine (DPA). The occurrence of autoantibodies was also described, the presence of which was not related to the type of treatment or the form of the disease (hepatic vs. neuropsychiatric). The mechanisms responsible for the occurrence of autoantibodies in patients with WD are not known. It has also not been clarified whether they have clinical significance. In some patients, WD was differentiated or coexisted with an autoimmune disease, including autoimmune hepatitis or multiple sclerosis. Various molecular mechanisms may be responsible for immunological abnormalities and/or the inflammatory processes in WD. Their better understanding may be important for explaining the reasons for the diversity of symptoms and the varied course and response to therapy, as well as for the development of new treatment regimens for WD.

## 1. Introduction

Wilson’s disease (WD) [Online Mendelian Inheritance in Males, OMIM #277900] is a rare disorder of copper metabolism [1]. The disease is inherited in an autosomal recessive manner. Its occurrence is due to a mutation in the *ATP7B* gene located on the long arm of chromosome 13. As a result, the function of ATPase 7B, encoded by *ATP7B*, an enzyme found mainly in liver cells and responsible for cellular copper transport, is impaired. It cannot cooperate with its partner, antioxidant protein 1 (ATOX1), which is necessary for the proper transport of copper in the cell; its enzymatic activity decreases, its half-life shortens, and/or it undergoes incorrect localization in the cell [2,3]. The incorrect function of ATPase 7B leads to copper incorrectly binding to apoceruloplasmin, and the excretion of excess copper in bile is impaired, which leads to copper retention in cells, primarily in hepatocytes. After exceeding the capacity threshold of liver cells, they are damaged, and copper is released into the bloodstream, where it remains unbound with ceruloplasmin, (CPN) and in this form it is very toxic, and it also easily accumulates in other organs, e.g., in the intestines, brain, kidneys, and cornea [1,2,3].

WD is present all over the world. The incidence of this disease in the general population is estimated at approximately 1:30,000. The disease is more common in closed populations with low population migration and/or consanguineous marriages; this includes China, Japan, the Canary Islands, Corsica, and Sardinia [4].

The clinical picture of WD is diverse. The first symptoms usually appear between the ages of 10 and 40. However, cases of an 8-month-old boy with an enlarged liver and patients whose symptoms of bipolar disorder appeared after the age of 60 were described [5,6,7].

Liver symptoms most often appear earlier, in the first and second decade of life, while neurological and/or psychological symptoms appear later. However, the course of the disease may vary, even in members of the same family or in identical twins. Tissue damage in WD may result from various molecular mechanisms. Oxidative stress, mitochondrial damage, and apoptosis play a particular role, but inflammatory/ immune/ autoimmune responses may also be important.

In 1976, Członkowska and Milewski described immunological changes in patients with WD for the first time. They observed an increased humoral immune response, i.e., higher levels of IgG and IgM, a higher titer of antibodies against the Kunin’s CA antigen, and decreased cellular immunity, i.e., a lower response against 2,4-dinitrochlorobenzene (DNCB) and E. coli, lower lymphocyte transformation after concanavalin stimulation A (Con A), purified tuberculin protein derivative (PPD), *Candida albicans*, and streptokinase, and lower production of macrophage migration inhibitory factor. They also found that leukocytes from WD patients had reduced bactericidal activity and that copper ions had an inhibitory effect on some cellular immunity tests. They hypothesized that immune disorders in WD result from liver cirrhosis, but the inhibitory effect of copper ions on the immune response and the related effect on leukocyte metabolism cannot be ruled out [8].

In the following years, many observations were published regarding various immune abnormalities in patients with WD. Changes in serum cytokine concentrations, the relationship between cytokine gene variants and the clinical course of the disease, the occurrence of various autoantibodies, the co-occurrence of autoimmune diseases, and autoimmune complications during treatment with preparations that reduce copper load in the body are described.

We aim to summarize the current knowledge on clinical and molecular aspects of inflammatory/immune/autoimmune responses in WD.

## 2. Wilson’s Disease—Clinical Presentations

### 2.1. Liver Disease

Liver symptoms in WD may vary from subtle inflammation to fulminant liver failure. The most common clinical symptoms are fatigue, jaundice, ascites, and hepatosplenomegaly. They usually appear in the second to third decade of life [9].

Some patients may only have transient symptoms resembling an acute viral infection. Transient jaundice with hemolytic anemia often occurs without signs of acute liver damage. Such episodes are often not precisely diagnosed and are reported by patients only when, often years later, neurological disorders appear, or liver failure occurs. Many patients secretly develop symptoms of compensated or uncompensated cirrhosis. Often the first symptom to cause suspicion of liver damage is spleen enlargement.

The most severe form of liver damage is fulminant liver failure, often with hemolytic anemia, thrombocytopenia, coagulopathy, renal failure, and encephalopathy. Symptoms of the disease may develop within a few days, most often without an identifiable cause, but may be provoked, for example, by contact with chemical substances or pregnancy [10]. It is anticipated that hepatocyte mitochondrial injury is the first step of copper-induced liver disease. Deformation of mitochondria, elongation, presence of inclusions, and cristae dilatations are observed. On a functional level, oxidative damage and high mitochondrial reactive oxygen species production are noted [11]. Mitochondrial dysfunction triggers inflammation, which, in turn, causes morphological changes of liver architecture [12]. Steatosis accompanied by glycogenated nuclei, Mallory bodies, inflammation, and fibrosis are documented in liver bioptates of WD patients [13]. Copper deposits in lysosomes, visualized with rodanine staining, are observed, but their distribution is not uniform in the liver parenchyma, and they are not present in every WD patient [14]. Rodanine-positive deposits are not characteristic of WD, as they are also seen in patients with cholestatic diseases. Without treatment, liver disease progresses to chronic hepatitis, necrosis, subsequent stages of fibrosis, and finally, cirrhosis, with all complications [14].

### 2.2. Central Nervous System (CNS) Disease

The neurological symptoms of WD usually appear later than the liver symptoms [15,16,17]. The dominant neurological symptoms in patients with WD are tremors, mainly postural, kinetic, or at rest; the typical postural tremor is often described as “wing beating”. Another common symptom is dystonia presenting as segmental, multifocal, or even generalized; sometimes very severe, even leading to contractures. Parkinsonism is also very common and may also be the first neurological symptom in WD, including disturbances in muscle tone, especially stiffness; bradykinesia (hypomimia); salivation; micrographia; and gait disturbances (lack of balance, shuffling). Cerebellar symptoms are also seen, including speech and tremors (intentional) and gait and balance disturbances. Dysarthria and dysphagia seem to be the most common neurological symptoms in WD; this is associated with damage to the extrapyramidal system and the cerebellum. Neurological symptoms are often accompanied by psychiatric symptoms (cognitive and/or behavioral or even psychotic syndromes); therefore, the term “neuropsychiatric symptoms of WD” is often used [15].

The structures most sensitive to the toxic effects of copper in the central nervous system are the substantia nigra, deep gray matter, and the red nucleus, due to their rapid metabolism [18]. On magnetic resonance imaging (MRI), lesions are most often observed in the putamen (64–86%), caudate nucleus (33–67%), globus pallidus (38–81%), thalamus (54–60%), midbrain (41–77%), and cerebellum (12–50%) [18].

A relationship has been demonstrated between the severity of the disease and the degree of changes on MRI. Typical morphological changes in glia are observed in WD [19,20]. This happens because astrocytes take up copper, which causes them to swell, hypertrophy, and multiply, ultimately transforming into giant cells with a characteristic morphology. In WD, the most typical are AIA and AIIA (Alzheimer’s type I and II) cells and Opalski (OPA) cells. AIA cells are large and multinucleated. They are distinguished by an eosinophilic cytoplasm and a large acinar nucleus. AIIA cells are characterized by the absence of cytoplasm around large nuclei, with low chromatin content. OPA cells are usually larger than neurons, round or oval, and have eosinophilic cytoplasm. Their nucleus is disproportionately small and hyperchromatic. The presence of all three cell types indicates impaired astroglial function and, if present in brainstem structures, may result in rapid clinical deterioration.

### 2.3. Other Symptoms

Almost all patients with neurological diseases have a Kayser–Fleischer (KF) ring, which is the result of copper accumulation in the Descemet corneal membrane.

Depending on the dominant symptoms, various clinical forms of WD are distinguished, including hepatic (approx. 45% of patients), neurological (approx. 35% of patients), and psychiatric (approx. 10% of patients). Five percent of patients also experience non-specific symptoms, such as bone and joint pain, kidney failure, irregular menstruation, and spontaneous miscarriages. Rarely, the first symptom of the disease is hemolytic anemia, Fanconi syndrome, pancreatitis, hypothyroidism and parathyroidism, or cardiomyopathy [1,2,3].

## 3. Mechanisms of Tissue Damage in WD

### 3.1. Oxidative Stress, Cuproptosis and Ferroptosis

Several hypotheses have been proposed as to the exact mechanism by which copper ions cause cell death. These include the initiation of apoptosis, caspase-independent cell death, generation of reactive oxygen species (ROS), and disruption of the ubiquitin–proteasome system. However, there is still no universally accepted theory. Therefore, further studies are needed to uncover the exact mechanisms controlling copper-induced cell death [21]. Interestingly, in WD not only copper, but also iron metabolism is affected. This is because the metabolic pathways of these two elements are related at several stages. For example, ceruloplasmin, a protein whose maturation, function, and half-life depends on copper, plays an important role in iron metabolism. Many studies have shown that these two elements accumulate in the tissues of patients with WD [22,23,24,25].

Copper and iron are redox-active and participate in the Fenton and Haber–Weiss reactions, which lead to the formation of free oxygen radicals. In these reactions, divalent Cu^2+^ is reduced to monovalent Cu^+^, which is converted back to Cu^2+^, while Fe^3+^ is reduced to Fe^2+^, which is converted back to Fe^3+^ [26]. These reactions intensify liver as well as CNS damage through an irreversible chain reaction mechanism. ROS disrupt the double helix structure of DNA, causing cell death [27]. ROS also damage the mitochondrial inner membrane, impair mitochondrial energy chain transport, and damage the mitochondrial DNA structure. This leads to abnormal mitochondrial function and reduced ATP production, leading to accelerated cell death [28]. In the Long–Evans cinnamon rat, an animal model of WD characterized by copper overload, copper accumulates in the mitochondria. This accumulation damages the integrity of the mitochondrial membrane, depletes glutathione (GSH) stores, and increases the damage of oxidative stress to organelles [29,30]. Third, ROS bind unsaturated fatty acids on the cell membrane. This activates lipid peroxidation and increases the permeability of the cell membrane, thereby causing cell death [31].

The results of our studies on the role of oxidative stress and natural antioxidant mechanisms in WD indicate that:

1. The effectiveness of antioxidant mechanisms is greatly reduced due to copper overload;
2. Treatment that reduces the burden of copper improves the antioxidant status of patients but does not resolve it;
3. The effectiveness of antioxidant protection increases after treatment;
4. Interindividual variability within genes encoding proteins involved in the antioxidant defense system may modulate phenotypic expressions of WD [32,33,34].

In addition to participating in free radical reactions, copper and iron can contribute to cell death in the cuproptosis and ferroptosis processes.

Cuproptosis is a relatively recently described form of cell death caused by copper accumulation in mitochondria, which impairs mitochondrial respiration and protein lipidation, leading to membrane permeability, cell damage, and the initiation of apoptosis pathways under various conditions [35,36,37,38]. Cuproptosis mainly occurs in energy-producing cells, which use oxidative phosphorylation (OXPHOS) as their main metabolic pathway, such as liver and CNS cells, which are most damaged in WD [21]. In the process of cuproptosis, excessive intracellular copper activates cell death pathways involving the mitochondrial ferredoxin 1 (FDX1) protein. Cuproptosis is characterized by the aggregation of mitochondrial lipoylated proteins, mainly lipoylated dihydrolipoamide S-acetyltransferase (DLAT) [21,39,40,41]. FDX1 serves as a controller of protein lipoylation, contributing to protein acylation, which determines copper binding to DLAT (component of the tricarboxylic acid (TCA) cycle) and subsequent acylation-dependent DLAT oligomerization. Abnormal DLAT oligomerization induces DLAT changes, which contribute to the formation of insoluble DLAT, protein toxicity, and cell death. FDX1 acts also as a reductase and converts Cu^2+^ to the more dangerous Cu^+^ form [42,43]. Excess Cu^+^ binds to lipoylated DLAT and further leads to DLAT oligomerization, which may lead to the initiation of copper-induced cell death.

In addition to the copper-induced accumulation of lipoylated mitochondrial DLAT enzymes, there is also a loss of iron–sulfur (Fe-S) cluster proteins, leading to proteotoxic stress [21,39,40,41]. Fe–S clusters are essential protein cofactors involved in various biological processes, including enzyme catalysis, electron transfer, and metabolic stress sensing. Since Fe–S clusters play a key role in enzymatic processes, protein function largely depends on the formation of iron–sulfur clusters through mitochondrial biogenesis. Fe–S biogenesis takes place on the inner mitochondrial membrane. Accumulated copper can destabilize Fe–S clusters, causing reduced expression of several key metabolic enzymes, putting the cell into a toxic state of stress that ultimately leads to death.

The cuproptosis ability of cells has been shown to be related to glutaminase (GLS) and metal regulatory transcription factor 1 (MTF1) [44]. GLS mainly stimulates glutamine catabolism, which converts glutamine to glutamate, which is a precursor for GSH synthesis [45,46]. MTF1 is a transcriptional regulator of cellular adaptation to heavy metals that activates the transcription of metallothionein (MT), a copper-binding protein, by binding to a metal response element in the MT promoter [47]. Thus, GLS and MTF1 may influence the susceptibility of cells to cuproptosis by influencing the intracellular levels of the GSH and MT [43].

GSH can bind intracellular copper, reducing its levels, preventing the accumulation of lipoylated proteins, and reducing Fe–S cluster proteins, ultimately preventing cuproptosis [48]. A decrease in GSH levels was demonstrated in patients with WD, confirming the involvement of the cuproptosis mechanism in the pathogenesis of cell damage caused by copper accumulation [32].

Another important mechanism in the pathogenesis of cell death in WD is ferroptosis [49]. Ferroptosis is an iron-dependent phospholipid peroxidation process that represents a spontaneous form of cell death that results from imbalances in cellular iron homeostasis, cellular metabolism, redox states, and the accumulation of lipid peroxides in the cell membrane, which leads to cell death [50,51]. Ferroptosis occurs in three main ways: iron accumulation, lipid peroxidation (LPO), and cystine degradation. Increasing the iron load is important for the induction of ferroptosis [50].

Dysregulated lipid metabolism, especially of fatty acids (FAs), is recognized as a key factor in the pathogenesis of ferroptosis [52,53]. Interestingly, monounsaturated fatty acids (MUFAs) and polyunsaturated fatty acids (PUFAs) have different effects on ferroptosis, which may be related to their chemical composition and stability, which, in turn, determines their effects on ferroptosis [37,38].

The balance between MUFA-phospholipid (MUFA-PL) and PUFA-PL plays an important role in determining the susceptibility to ferroptosis [54,55]. In this regard, acetyl coenzyme A (acetyl-CoA) is converted to malonyl-CoA and PUFA is produced by the action of acetyl-CoA carboxylase (ACC). Next, acyl-CoA synthetase 4 (ACSL4) and long-chain lysophosphatidylcholine acyltransferase 3 (LPCAT3) promote the conversion of PUFA to PUFA-PL [49]. Under the influence of iron-dependent lipoxygenase and ROS, PL-PUFA undergoes peroxidation and produces polyunsaturated phospholipid hydroperoxide (PUFA-PL-OOH), thereby causing ferroptosis [54,55,56].

Previous studies have shown that glutaminolysis is required for cysteine deprivation-induced ferroptosis [57,58]. Glutaminolyase, which is essential for cysteine deprivation-induced ferroptosis, acts as an anaplerotic pathway for the mitochondrial TCA cycle and promotes ferroptosis by increasing lipid peroxidation [58]. In addition, the activity of the mitochondrial electron transport chain (ETC) has been linked to the regulation of cysteine deprivation-induced ferroptosis, which affects mitochondrial membrane potential, lipid peroxide accumulation, and the susceptibility of the cell to ferroptosis [58]. The mitochondrial TCA cycle and the ETC play an important role in the initiation of ferroptosis by increasing mitochondrial membrane potential, hyperpolarization, and lipid peroxide accumulation [58].

In both types of cell death, ferroptosis and cuproptosis, mitochondria play an important role. Ferroptosis and cuproptosis are closely related to mitochondrial metabolism [21,36], highlighting the importance of studying mitochondria to understand mechanisms, regulatory processes, and disease implications.

Cuproptosis is associated with visible changes in mitochondrial morphology, such as shrinkage and membrane rupture [59]. Mitochondrial respiration and enzymes play an important role in the regulation of cuproptosis, showing its dependence on mitochondrial respiration [60]. The mitochondrial TCA cycle plays an important role in the occurrence of cuproptosis, in which protein lipoylation is specific to the four proteins: dihydrolipoamide branched-chain transacylase E2 (DBT), glycine cleavage system protein H (GCSH), dihydrolipoamide S-succinyltransferase (DLST), and DLAT, involved in this cycle [60]. Copper cytotoxicity impairs mitochondrial integrity by the accumulation of lipoylated proteins and causing the loss of Fe–S cluster proteins, resulting in cuproptosis [60,61]. The main morphological signs of cuproptosis are mitochondrial degeneration, cell membrane damage, endoplasmic reticulum damage, and chromatin damage, similar to apoptosis.

Ferroptosis is characterized by specific morphological changes in mitochondria, including volume reduction, increased membrane permeability, disruption of the mitochondrial outer membrane, and loss of mitochondrial cristae [62,63]. In addition to morphological changes, ferroptosis affects mitochondrial function, leading to reduced ATP synthesis, DNA damage and reduced heme synthesis [63,64,65,66]. Ferroptosis includes mitochondrial morphological changes, such as shrinkage and increased membrane and mitochondrial fragmentation [67,68], which are not observed in cuproptosis.

From the above, it can be concluded that the mitochondrial TCA cycle is the common point of ferroptosis and cuproptosis. The DLAT protein, which is closely related to the TCA cycle, is lipoylated by FDX1, and the combination of the lipoylated protein with copper triggers cuproptosis [21].

In addition to the mitochondrial TCA cycle, GSH acts as a common site for ferroptosis and cuproptosis, although its functions are different [21]. In ferroptosis, GSH acts as an antioxidant by thwarting LPO, while in cuproptosis, it acts as a copper chaperone, binding copper to alleviate the aggregation of lipoylated proteins [65,69]. Interestingly, GSH exhibits inhibitory effects on both ferroptosis and cuproptosis, suggesting a co-regulatory relationship in which GSH may play a key role in mediating crosstalk between these processes.

It is worth mentioning that disruption of copper homeostasis not only regulates ferroptosis and cuproptosis but also triggers autophagy [70]. Intracellular excess copper can activate transcription factor EB (TFEB), upregulate the expression of autophagy protein 5 (ATG5), sequestosome 1 (SQSTM1), and microtubule-associated light protein 3 (MAP1LC3) 1, and regulate the AMP-activated protein kinase–mechanistic target-of-rapamycin (AMPK-mTOR) pathway to induce autophagy [70]. Activation of autophagy, especially selective forms, such as ferritinophagy, lipophagy, and clockophagy, is crucial for the initiation of ferroptosis [71,72,73]. In relation to ferroptosis, autophagy acts by degrading anti-ferroptosis factors, such as ferritin, lipid droplets, and glutathione peroxidase 4 (GPX4) [71,72,73,74].

### 3.2. Inflammation

The inflammatory process is part of the protective response to various tissue injuries and is usually a beneficial process [75,76]. Unfortunatelu, uncontrolled chronic inflammation may contribute to a variety of chronic inflammatory diseases [77]. Various factors, including infections, cell damage, toxins, radiation, and free radical damage, can influence the development of inflammation [77]. Copper not only increases the concentration of free radicals in the cells, but also weakens the ability to eliminate them by reducing the amount of the antioxidant GSH [78]. During an inflammatory reaction, immune cells also play a role in increasing oxidative stress by producing superoxide anions and nitric oxide (NO). These reactions result in DNA fragmentation and lipid oxidation, as well as the release of additional copper ions, which increase oxidation [78]. It has been shown that copper can activate transcription factors sensitive to oxidative factors, which can promote the production of inflammatory mediators in endothelial cells [78]. Uncontrolled production of ROS and/or reactive nitrogen species (RNS) can cause the activation of transcription factors, including activator protein-1 (AP-1), hypoxia-inducible factor-1α (HIF-1α), and nuclear factor-κB (NF-κB); all these factors are involved in inducing an inflammatory response [79,80]. Of these, the most important role is played by NF-κB, which participates in the recruitment and activation of cells of the immune system by inducing the production of cytokines, chemokines, and adhesion molecules [81].

In vivo studies have shown that chronic exposure to excessive copper concentrations promotes inflammatory and oxidative processes in the CNS of laboratory animals [82]. Excess extracellular copper has been shown to contribute to neuronal damage by increasing the production of free radicals. Other molecular mechanisms of copper neurotoxicity remain weakly recognized. Increased extracellular copper has been shown to strongly influence the secretion of interleukin-1α (IL-1α), IL-12, and RANTES (regulated upon activation, normal T cell expressed and secreted). Copper was able to induce GN11 and primary neurons into an inflammatory state [83]. Copper has been reported to induce IL-6 in a cell culture system consisting of human keratinocytes and fibroblasts [84]. Medici et al. found that excess hepatic copper was associated with inflammation and increased serum alanine aminotransferase (ALT) and hepatic tumor necrosis factor-α (TNF-α) [85].

**Figure 1:**
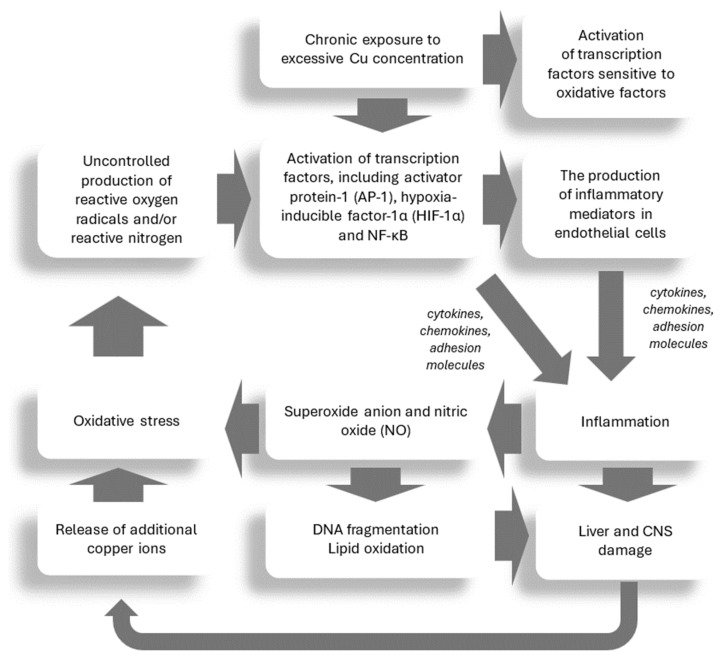
Copper–oxidative stress–inflammation relationships in the pathogenesis of liver and CNS damage. CNS, central nervous system; NF-kB, nuclear factor-kappa B.

Therefore, it may be hypothesized that cytokine-mediated inflammation might be important in the modulation of liver and CNS tissue damage in WD (see: Figure 1). More information on this topic can be found in the following chapters.

#### 3.2.1. Copper-Associated Inflammation and Liver Pathology in WD

The pathogenesis of liver cell damage from copper overload is not fully understood. A common feature of WD is the presence of liver inflammation, ranging from chronic hepatitis to fulminant hepatitis [1,5]. Liver inflammation in WD is closely related to copper accumulation.

In an experimental study with an animal model of WD using C3HeB/FeJ-Atp7btx-J/J (tx-j) mice, changes in hepatocyte morphology and increased hepatocyte to nuclear diameter and nuclear to hepatocyte diameter ratios were observed. Nuclear and cellular enlargement of hepatocytes, which is often associated with chronic hepatitis with enzymatic activation of hepatocytes, has been previously described in WD patients and animal models of the disease [1,5,14,86,87,88,89]. Early stages of liver impairment in WD have been shown to include portal inflammation, which may be characterized by lymphocytic and neutrophilic infiltration, and microvesicular and macrovesicular steatosis [9,89].

Different types and symptoms of non-infectious hepatitis are seen in WD. Histologically, liver pathology presents with acute or chronic inflammation, sometimes mimicking inflammation associated with viral infection, autoimmune disease, or other causes of liver damage. Inflammatory infiltrates can invade the portal spaces, sometimes accompanied by periportal necrosis and endplate destruction of periportal hepatocytes by inflammatory cells (vital necrosis). Inflammatory cells can also be seen in intralobular areas, causing damage and necrosis of small groups of hepatocytes. Both portal and lobular hepatitis can coexist in individual patients [89].

Medici et al. [85] found that excess hepatic copper is associated with inflammation, manifesting as pathological changes in the organ and increased serum levels of ALT and TNF-α. Copper can activate NF-κB signaling in the liver and thus stimulate the synthesis of pro-inflammatory cytokines [90,91]. It has been shown that copper induces the production of IL-6 in fibroblasts and keratinocytes, and in various studies it was documented that the intensity of the inflammatory response is related to the systemic/ tissue concentration of copper [92,93,94,95].

The results of a study by Ho et al. confirmed that *Atp7b*^−/−^ mice develop hepatic phenotypes of WD, with increased liver parenchymal enzymes, impaired lipid metabolism, hepatosplenomegaly, and liver inflammation and fibrosis [96].

Interestingly, the results of a study by Medici et al. suggest that an important link mediating the relationship between inflammation and liver damage in WD is changes in methionine (Met) metabolism [85].

Previous studies have shown that Met deficiency is associated with copper overload in the liver [97,98]. Impairment of Met metabolism is associated with epigenetic regulation of gene expression [99]. It is important for the regulation of S-adenosylhomocysteine (SAH), which prevents methylation reactions and can sensitize hepatocytes to the presence of TNF-α. At the same time, the inflammatory process—promoting increased cell division and DNA repair—increases the need for methylation [100,101]. SAH is the substrate for the bivalent SAH hydrolase (SAHH). Copper can regulate Met metabolism through its inhibitory effect on SAHH, and, consequently, an increase in SAH, the main inhibitor of the transmethylation reaction [97,98,102]. In untreated tx-j mice, the transcript levels of selected genes related to endoplasmic reticulum (ER) stress, lipogenesis and β-fatty acid oxidation, and sterol regulatory element-binding proteins (SREBP1c) and peroxisome proliferator-activated receptor alpha (PPARα) protein levels, was reduced. Liver SAH levels were increased, and SAHH gene and protein expression, as well as SAM to SAH ratio were decreased. DNA methyltransferase 1 (Dnmt1) transcripts were upregulated, Dnmt3a transcripts were similar to controls, and Dnmt3b transcripts were downregulated. Global DNA methylation was lower in tx-j mice than in the control group. It has been shown that copper chelation of penicillamine (PCA) reduced inflammation in tx-j mice, decreased the expression of TNF-α and selected genes related to ER stress and lipid metabolism, and normalized global DNA methylation levels. This was associated with better lobular and portal absorption [85]. The authors of the study suggest that the interplay between inflammation and Met metabolism is related to copper-mediated inhibition of SAHH, resulting in elevated levels of SAH, which dysregulates methylation status and gene expression in WD.

Currently, the molecular mechanisms linking copper and gene expression are unknown, but it seems likely that methylation plays an important role. Decreased global methylation in tx-j mice may be associated with increased inflammation, which others have linked to an increased demand for gene methylation resulting from increased cell and DNA division [100,101]. Therefore, WD may be associated with increased demand for methyl groups because of both an increase in SAH methylation inhibitor and increased inflammation.

The regulatory role of increased copper in downregulating SAHH activity, and the consequent upregulation of its substrate SAH and its potential secondary epigenetic effects on gene expression, suggest that Met metabolism may be the missing link between copper accumulation, inflammation, and hepatocyte damage in WD.

Interesting research results on the relationship between copper, inflammation, and liver damage in WD were published by Ho et al. [96]. The authors showed that *Atp7b^−/−^* knockout mice had increased numbers of CD45^++^ immune cells and F4/80^++^ macrophages in the liver, as well as p65 upregulation in the liver. As part of nuclear factor kappa activation of the light chain of the activated NF-κB pathway, cellular expression of p65 is involved in the inflammatory process. Immunohistochemical analysis showed a significant increase in the number of p65^+^ cells in the liver in the *Atp7b^−/−^* group compared to the *Atp7b^+/−^* control group. Alteration of p65 in copper-loaded WD livers is associated with changes in the cellular trafficking of the negative molecular pattern protein (danger-/damage-associated molecular patterns, DAMPs); high-mobility group box 1 (HMGB-1). HMGB1 is a non-histone nuclear protein that has many functions according to its subcellular location. In the nucleus, HMGB1 is a DNA chaperone that maintains the structure and function of chromosomes. In the cytoplasm, HMGB1 can promote autophagy by binding to the beclin-1 (BECN1) protein.

Cellular copper accumulation has been shown to induce depletion of ATP, which activates AMP-activated protein kinase (AMPK), resulting in HMGB1 phosphorylation and increased extracellular release. After active secretion or release, extracellular HMGB1 mainly acts as a DAMP molecule and regulates inflammation and the immune response through various receptors or direct uptake [103].

Increased hepatic expression of HMGB1 in *Atp7b^−/−^* mice was related to splenic CD11b^+^/CD43^+^/Ly6C^Hi^ inflammatory monocyte expansion that contributes to splenomegaly and elevated circulating levels of proinflammatory cytokines. Indeed, HMGB-1 is both passively released by necrotic cells or actively secreted by activated cells and leads to downstream signaling of NF-κB activation via p65 and thus proinflammatory cytokine production [96].

Ho et al. proved that liposome-encapsulated curcumin (LEC) management keeps HMGB1 within the nucleus of *Atp7b^−/−^* mice and, as a result, prevents the downstream expression of p65. This eventually decreases hepatic infection and splenic CD11b^+^/CD43^+^/Ly6C^Hi^ inflammatory monocytes and decreases circulating levels of proinflammatory cytokines in WD. These effects contribute to the prevention of hepatic fibrosis and attenuation of hepatosplenomegaly by LEC in WD. Importantly, the reduced local hepatic and systemic inflammatory reactions following LEC led to the attenuation of liver harm and fibrosis, independent of the modulation of copper content material within the liver in WD. The authors conclude that treatment with subcutaneous LEC may also ameliorate copper-triggered liver injury in an animal model of WD through suppressing HMGB1-mediated hepatic and systemic inflammation [96].

It is worth mentioning here that the production of HMGB1 in WD may be related not only to the up regulation of p65, but HMGB1 secretion may be stimulated through many factors, including those that may be important from the point of view of the mechanism of tissue damage in WD, such as oxidative stress, inflammation, apoptosis, necroptosis, cuproptosis, and ferroptosis. Copper accumulation-triggered damage to the mitochondrial respiratory chain and ATP depletion turn on AMPK to promote cuproptosis and HMGB1 phosphorylation, resulting in elevated extracellular release [104].

HMGB1 is considered a conventional pro-inflammatory mediator, among other functions, due to the fact that it could activate immunocompetent cells and stimulate pro-inflammatory cytokines, together with TNF-α, IL-1, and others, and may contribute to the induction of fever and anorexia. Its actions are synergistically enhanced in the presence of exogenous toll-like receptor (TLR) agonists and other pro-inflammatory cytokines, and this may be specifically targeted for therapeutic benefit in disease syndromes associated with increased levels of HMGB1.

It has also been shown that HMGB1 is directly involved in the positive regulation and maintenance of ferroptosis in cells, possibly through the regulation of iron-mediated lipid ROS production. Depletion of HMGB1 inhibits lipid peroxidation and reduces iron accumulation in cells [105].

#### 3.2.2. Copper-Associated Inflammation and CNS Pathology in WD

After the liver, the brain is the second organ that accumulates the most copper [82,106]. The highest concentration of copper in the systemic circulation was found in the substantia nigra [82]. In patients with neuropsychiatric-type WD, copper in cerebrospinal fluid (CSF) and brain tissue reaches 85% of normal CNS levels. It increases even after years of treatment with chelation [107]. It was shown that the severity of neuropathological findings is related to the concentration of copper in the brain [108].

Previous studies have shown that copper accumulates in the striatum in mouse models of WD and increases IL-6, IL-8, IL-10 and TNF-α in this region. The inflammatory response in different areas was affected by the amount of copper accumulation [109].

In a study by Kalita et al., patients with WD have been shown to have higher levels of malondialdehyde (MDA), glutamate, and cytokines (IL-6, IL-8, IL-10 and TNF-α), and lower levels of GSH and weaker total antioxidant capacity (TAC) compared to the control group. Serum glutamate, IL-6, IL-8, and malondialdehyde increased with increasing neurological severity, whereas GSH and TAC decreased [110]. These data confirm the relationship between oxidative stress, inflammation, and neurodegeneration in WD. Wu et al. found a significant increase in T helper (Th) 1 cells (IL-2, TNF-α, and TNF-β), Th2 cells (IL-5, IL-10, and IL-13) and Th17 (IL- 23) (*p* < 0.05). Higher levels of plasma Th 1 cells (IL-2, TNF-α, and TNF-β), Th2 cells (IL-13), and Th 17 cells (transforming growth factor-β1 (TGF-β1) and IL-23) were observed in in neurological patients compared to controls (*p* < 0.01). In addition, the number of Th 1 (TNF-α and TNF-β), Th 3 (TGF-β1), and Th 17 (IL-23) cells were significantly higher in hepatic and neurological patients (*p* < 0.05). In addition, higher numbers of Th1 cells (IL-2, TNF-α and TNF-β), Th2 cells (IL-13), and Th17 cells (TGF-β1, IL-23) were associated with more severe neurological symptoms of WD [90].

The above findings suggest that the increase in copper concentration in the CNS is related to oxidative stress and neuroinflammation, which affects the microenvironmental homeostasis in the CNS. The human body’s ability to deal with increased inflammatory cytokines may be a factor that determines the severity of neurological symptoms of WD.

### 3.3. Pro- and Anti-Inflammatory Cytokine Gene Polymorphisms and the Phenotype of WD

In humans, large interindividual variability has been observed in the production of cytokines in normal and pathological conditions. This variability is genetically determined—it is related to the occurrence of functionally important polymorphisms in genes encoding cytokines. Individual alleles of genes encoding cytokines are associated with increased or decreased production of cytokines in normal and/or pathological conditions.

This knowledge became the basis for our hypothesis that genetically determined variability in the synthesis of pro- and anti-inflammatory cytokines may be one of the factors modifying the phenotypic picture of WD.

It has been shown that the polymorphism of the cytokine genes’ *IL1B* C-511T and *IL1RN* (the gene encoding the IL-1 receptor antagonist (IL-1RA)) variable number of tandem repeats (VNTRs) is associated with copper metabolism in patients with WD [111]. Carrying the *IL1RN* VNTR*2 allele favors the earlier onset of clinical symptoms of WD, especially in patients with the neuropsychiatric form of the disease. Carrying the *IL1B* -511T allele is associated with higher concentrations of copper and ceruloplasmin in the serum of patients with WD. This relationship can be explained by the activity of IL-1β as one of the factors inducing the so-called acute phase reaction, which is associated with the increased synthesis of acute-phase proteins in the liver, ceruloplasmin being one of these proteins. The observation of increased copper concentration in carriers of the *IL1B* -511T allele is probably directly related to the effect of IL-1β on the increase in ceruloplasmin production [111].

An association of the IL1RN*2 allele with increased serum ceruloplasmin levels was also reported (this observation applies only to patients without the p.H1069Q mutation in one or both *ATP7B* alleles). The same allele was associated with earlier (by 3.5 years) manifestation of the first symptoms of WD. In the group of patients with the *IL1B* -511T allele, the difference in the age of WD patients at the time of the first symptoms of WD between carriers and non-carriers of the IL1RN*2 allele was 8 years (23 years versus 31 years, respectively). The influence of the IL1RN*2 allele on the earlier clinical manifestation of WD was significant in the group of patients with neuropsychiatric WD [111].

The authors interpreted their observations, taking into account many aspects regarding the phenotypic effects of polymorphic variants of cytokine genes. Evidence has been presented that suggests that the phenotypic effect associated with genetic variability in pro- and anti-inflammatory cytokine genes is determined by the presence of specific haplotypes, characterized by the coexistence of specific variants of individual cytokine genes, and not by the genotype of a single locus [112]. Observations regarding the variability of phenotypic effects determined by the haplotype regarding the simultaneous presence of specific polymorphic variants of the *IL1RN* and *IL1B* genes confirm such a hypothesis.

When interpreting the obtained research results, it was noted that, in some studies, the influence of the genotype concerning polymorphic variants of post-inflammatory cytokine genes on the risk/age of patients at the time of occurrence of neurodegenerative diseases depended on the ethnic group (for example, the influence of the *IL1B*-511TT genotype on the increased risk of Alzheimer’s disease (AD) in the Caucasian population [113]), or on the genetic background related, for example, to the apolipoprotein E (*APOE)* genotype (for example, the *TNF*-308T allele was associated with an increased risk of AD among non-carriers of the *APOE* allele system ε4, and the *TNF* -850T allele was associated with an increased risk of AD in *APOE* ε4 carriers [114]). Gromadzka et al.’s observations regarding an ethnically homogeneous population of WD patients suggest that, in this population, the phenotypic effect of the *IL1RN* VNTR polymorphism depends on the genetic basis of the *ATP7B* genotype. Also, Gromadzka et al., did not note the impact of the tested genotypes on the risk of developing neuropsychiatric symptoms of WD, but a significant impact of the IL1RN*2 allele on the age of patients at the time of occurrence of these symptoms was observed [111].

## 4. Cuproptosis, Ferroptosis, and Impaired Immune Reactivity in WD

As it was mentioned above, in 1976, Członkowska and Milewski described immunological changes in patients with bipolar disorder for the first time. Especially, they observed decreased cellular immunity, lower lymphocyte transformation, and lower production of macrophage migration inhibitory factor. They also found that leukocytes from WD patients had reduced bactericidal activity. They hypothesized that immune abnormalities in WD result from liver cirrhosis but did not rule out the inhibitory effect of copper ions on the immune response [8].

Considering today’s knowledge, it seems likely that cuproptosis and processes related to damage to the mitochondrial respiratory chain play an important role in determining immunoreactivity in WD. Copper accumulation-induced damage to the mitochondrial respiratory chain and ATP depletion activate AMPK to promote cuproptosis and HMGB1 phosphorylation, resulting in increased extracellular release. HMGB1 may interact with many immune receptors, including receptors for advanced glycation end-products (RAGEs). While the HMGB1-RAGE axis drives inflammation, under certain microenvironmental conditions it can also induce immune tolerance by inducing anti-inflammatory macrophages [115].

Released HMGB1 promotes the accumulation of M2-like macrophages and an IL-10-rich environment through selective signaling through RAGEs [116]. It has been shown that complement component C1q can form a multimolecular signaling complex with HMGB1, RAGEs, and leukocyte Ig-like receptor (LAIR-1) in lipid rafts and inhibit inflammation by promoting M2-like macrophage polarization [117]. HMGB1 has also been shown to support leukotriene production and induce interferon regulatory factor 5 (IRF5) in a RAGE-dependent manner, while producing specialized pro-resolving lipid mediators (SPMs) that have a negative effect on leukotriene synthesis and help resolve inflammation [118,119,120].

The HMGB1–RAGE axis plays a role in regulatory T cells (Tregs), which are a subset of CD4^+^ T cells and suppress the T cell immune response and maintain immune tolerance.

Yang et al. found that CD163 is an anti-inflammatory receptor for HMGB1–haptoglobin complexes [121]. Haptoglobin is an endogenous HMGB1-binding protein that targets HMGB1 to the CD163-dependent receptor pathway that induces heme-oxygenase-1 (HO-1) and IL-10 in the monocyte–macrophage lineage [121]. Another study showed that binding of HMGB1 by soluble CD52, a glycophosphatidylinositol-anchored glycoprotein, promotes ligation of soluble CD52 with the sialic acid-binding Ig-like lectin-10 receptor and suppression of T cell function [122].

Another possible mechanism contributing to abnormalities in cellular immunoreactivity in WD may be related to ferroptosis. Numerous studies have shown that ferroptosis primarily modulates the immune response by regulating immune cell activity [123,124,125]. Within the tumor microenvironment (TME), immune cell subtypes, including T cells, B cells, granulocytes, and monocytes, undergo spontaneous ferroptosis, affecting the overall immune response [126]. Recent discoveries by the group of Kim et al. emphasize that immunosuppressive cells of myeloid origin (granulocytic/polymorphonuclear myeloid-derived suppressor cells, PMN–MDSCs) subjected to ferroptosis release lipid peroxides, inhibiting the activity of T lymphocytes [124].

In gastric cancer, long noncoding RNAs (lncRNAs) targeting ferroptosis-related genes attenuate CD4+ T cell activity and promote immune escape, leading to a poor prognosis [127]. In 2020, Florida Voli’s group showed that copper levels in cancer cells can influence the expression of programmed death-ligand 1 (PD-L1), a ligand for the programmed death receptor PD-1. Exogenous copper supplementation increases PD-L1 mRNA and protein levels in cancer cells [128]. The PD-1 receptor, first described by Ishida et al. [129], is expressed primarily on activated T and B lymphocytes, but also on activated monocytes, dendritic cells, and natural killer (NK) and NKT cells [130]. The expression of the PD-1 molecule, which is observed in many cell populations, in contrast to the limited occurrence of other molecules belonging to the CD28 superfamily of molecules to T lymphocytes, suggests that the PD-1 receptor plays a major role in the regulation of the immune response [131].

PD-1 expression is induced by the T cell receptor (TCR) and B cell receptor (BCR) signaling pathways and is maintained during antigen stimulation [132,133]. Moreover, some cytokines (IL-2, -7, -15, and -21), TLRs, and interferons can stimulate PD-1 expression on T cells [134]. PD-L1 expression can be induced on most cells of the human body [135].

The cytoplasmic domain of PD-1 contains two tyrosine-based immunoreceptor motifs: immunoreceptor tyrosine-based inhibition motif (ITIM) and immunoreceptor tyrosine-based switch motif (ITSM). Ligation of PD-1 with PD-L1 or PD-L2 inhibits the signal transmitted from activated T lymphocytes and reduces the expression of pro-inflammatory cytokines and anti-apoptotic molecules. This contributes to a state of immunosuppression [136].

Recent research shows that the microenvironment in which chronic inflammation takes place is largely like the tumor microenvironment. In both cases, phenomena related to the development of immunological tolerance and the “exhaustion” of immune system cells are observed [137].

“Exhaustion” of lymphocytes is a process resulting in the loss of their cytotoxic functions, which results in impaired immune response mainly in the course of chronic viral infections, i.e., hepatitis B, hepatitis C, and human immunodeficiency virus (HIV) infection, as well as cancer, malaria, and *Mycobacterium tuberculosis* infections [138]. The first sign of lymphocyte “exhaustion” is a decrease in the secretion of IL-2 and other cytokines, including TNF-α. Another feature of “exhausted” T lymphocytes is the impaired ability to proliferate after contact with the antigen and the loss of the ability to self-renew with the participation of IL-7 and IL-15 cytokines [138,139].

Another cause of immunodeficiency in WD may be liver cirrhosis [140] (remembering that it occurs only in some patients). Liver cirrhosis is associated with several abnormalities in the innate and adaptive components of the immune system’s response to microbial challenge, leading to an acquired immunodeficiency state. These abnormalities include: (i) damage to the immune surveillance function of the liver, associated with structural damage and/or damage to Kupffer cells, which contributes to impaired removal of endotoxins and bacteria and may lead to bacteremia and permanent stimulation of the immune system and even death, which has been observed in experimental studies [141,142]; (ii) impairment of the synthesis of innate immunity proteins and pattern recognition receptors (PRRs), which contributes to the reduced bactericidal ability of phagocytic cells; (iii) damage to circulating immune cells at the systemic level. So far, abnormalities have been described in the following areas: (a) reduced number of neutrophils and impaired phagocytosis [143,144,145,146]; (b) reduced number and impaired function of monocytes and their pro-inflammatory phenotype and impaired Fc-γ receptor function; (c) decreased B cell numbers and memory B cell dysfunction; and (d) lymphopenia and reduced T cell proliferation, affecting Th and cytotoxic (Tc) cells [147,148,149,150] resulting from: (i) impaired de novo production of new naïve T cells due to accelerated aging and thymic atrophy, (ii) reduction of the memory T cell subset due to spleen sequestration and cell consumption associated with, among other factors, increased apoptosis, and (iii) impaired compensatory peripheral proliferation [151,152,153,154]; (iv) weakening the reactivity of circulating NK cells [155]. 

## 5. WD—Autoimmunity

### 5.1. Autoimmune Events and Autoantibodies during Anti-Copper Treatment

WD can be effectively treated with pharmacological agents, thanks to which a negative copper balance can be achieved. Two groups of drugs are currently used: (1) chelates [DPA or trientine (TN)], which increase urinary copper excretion; and (2) zinc salts, which reduce the absorption of copper from the gastrointestinal tract [156,157]. Long-term studies have documented positive results in nearly 85 percent of properly treated patients. Effective treatment requires lifelong treatment, which requires continuous monitoring of effectiveness and side effects [1]. Failure to adhere to treatment may lead to disease recurrence and liver failure. Moreover, the success of treatment depends on early diagnosis, which may be difficult [158].

DPA, developed in 1956 by John Walshe, is still the most widely used treatment method, with probably the best and longest treatment history [159]. However, DPA may cause several potentially significant adverse reactions (ADRs) [160]. Therefore, the safety of DPA use should be carefully monitored not only at the beginning of treatment, but also during its duration.

The mechanism of action of DPA in causing autoimmune complications is still poorly understood. Two theories have been proposed: (i) modification of autoantigens due to the presence of a highly reactive thiol group and (ii) impaired cellular cooperation of suppressor or effector lymphocytes with associated lymphoid cells. It has also been documented that penicillamine binds to aldehydes on the surface of macrophages and leads to their activation, which may lead to an autoimmune syndrome in some patients [161]. Treatment of the murine macrophage cell line RAW264.7 with penicillamine increased the production of TNF-α, IL-6, and IL-23, providing further evidence that penicillamine activates macrophages. Increased macrophage-activating cytokines, interferon gamma (IFN-γ) and granulocyte–macrophage colony stimulating factor (GM-CSF), and decreased IL-13 levels indicated NK cell activation, suggesting a positive feedback loop between macrophages and NK cells. Moreover, some studies suggest that Th17 cells are involved in the pathogenesis of this penicillamine-induced autoimmunity [162]. DPA has been shown to stimulate oligoclonal B cell activity as well as the production of autoantibodies, including antinuclear antibodies (ANAs), antineutrophil cytoplasmic antibodies (ANCAs), anti-muscle-specific tyrosine kinase antibodies, and anti-acetylcholine receptor antibodies [163,164,165].

There have been published reports of patients with WD who experienced side effects related to autoimmune disease during DPA treatment.

Autoimmune complications caused by DPA can be divided into two groups: organ-specific diseases, such as myasthenia gravis, polymyositis, and thyroiditis, and non-organ-specific diseases, such as Sjögren’s syndrome and lupus [161]. Discontinuation of DPA usually results in resolution of symptoms, but treatment with corticosteroids and immunosuppressants is sometimes necessary.

In 1971, Harpey et al. described a woman with WD who, after more than a year of treatment with d-penicillamine, developed symptoms of systemic lupus erythematosus (SLE) and increased ANA titer [166]. After discontinuation of DPA and steroid therapy, the symptoms of SLE in this patient disappeared and did not recur even after discontinuation of steroid therapy [166]. Harpey hypothesized that DPA may bind to epidermal proteins containing significant amounts of cysteine and may act as a chemical hapten. This new antigenic determinant can initiate cellular sensitization to both the hapten and the carrier protein. Sensitized killer lymphocytes can cause cell death, with the release of cellular antigens, i.e., DNA, and then induce secondary sensitization [166]. Other cases of lupus-like syndromes caused by DPA were described in the 1970s and, in initial reports, their incidence was approximately 2% [160].

In 1981, Walshe described eight patients who, after DPA treatment, developed symptoms suggesting the diagnosis of SLE [167]. In 1995, a woman with arthritis after DPA was described [168]. In 2012, further women were described in whom symptoms suggestive of SLE appeared 2 years after DPA treatment [169]. The next patient described as suffering from DPA-related SLE was a 23-year-old male who developed SLE symptoms 11 years after starting DPA therapy [170]. We found four other papers describing SLE as a complication of DPA treatment; they were published in 1971 [171], 1985 [172], 1987 [173], and 1999 [174]. Unfortunately, these works were published in Spanish [173], French [171], and [174] and Slovak [172] and we did not have access to their full texts.

The first symptoms of the syndrome in the described patients usually appear 6–12 months after starting DPA therapy, and the risk factors are older age and female gender. The most common clinical symptoms are pleurisy, polyarthritis, leukopenia, thrombocytopenia, antinuclear antibodies (less often against histones), and lupus erythematosus. Clinical symptoms and laboratory test results (antibodies, abnormal cells) return after discontinuation of DPA. The current incidence of lupus-like syndromes induced by DPA appears to be much lower, but this is not known based on product characteristics [175].

Recently, Antos et al. described a patient with WD who was diagnosed with drug-induced lupus erythematosus (LE) (DIL) caused by DPA after a year of treatment [176]. The patient reported fever up to 39 °C with joint pain in the shoulders, hips, knees, feet, and thoracic spine, which led to impaired mobility. Chest X-ray revealed pleurisy with pleural effusion, an increased level of C-reactive protein (CRP) (161 mg/L, normal: 0–5 mg/L), and an erythrocyte sedimentation rate of 40 mm/h (normal: 0–10 mm/h); blood tests showed the presence of lupus anticoagulant and ANA. After discontinuing DPA, zinc sulfate was started, and after treatment with methylprednisolone, the symptoms gradually decreased and eventually disappeared. After a month, the ANA results were negative. During four years of follow-up, the patient had no symptoms of SLE [176].

Table 1 shows patients with WD diagnosed with SLE after treatment with DPA.

Additionally, WD cases with post-DPA SLE were described in four papers published in Spanish, French, and Slovak:

- Boudin G, Pépin B, Godeau P, Vernant JC, Gouerou H. Lupus érythémateux induit par la pénicillamine au cours d’une maladie de Wilson [Lupus erythematosus due to penicillamine associated with Wilson’s disease]. Ann Med Interne (Paris). 1971 Feb;122(2):269-73. French. [171]
- Kalina P, Procházková L, Hauftová D. Syndróm podobný systémovému lupus erythematosus vyvolaný penicilamínom u pacienta s Wilsonovou chorobou [A syndrome similar to systemic lupus erythematosus caused by penicillamine in patients with Wilson’s disease]. Bratisl Lek Listy. 1985 Sep;84(3):336-40. Slovak. [172]
- López-Guerra N, Alvarez Lario B, García-Moncó C, Peña Sagredo JL. Lupus eritematoso sistémico inducido por d-penicilamina en enfermedad de Wilson: a propósito de un caso [Systemic lupus erythematosus induced by d-penicillamine in Wilson’s disease: apropos of a case]. Med Clin (Barc). 1987 Apr 4;88(13):552-4. Spanish. [173]
- Jan V, Callens A, Machet L, Machet MC, Lorette G, Vaillant L. Pemphigus, polymyosite et myasthénie induits par la D-pénicillamine [D-penicillamine-induced pemphigus, polymyositis and myasthenia]. Ann Dermatol Venereol. 1999 Feb;126(2):153-6. French. [174]

Other reports have been published describing DPA-induced myasthenia gravis (MG) in patients with WD; these patients are briefly characterized in Table 2 [177,178,179,180,181,182,183].

Antos et al. described a 51-year-old man diagnosed with WD based on abnormal copper metabolism, liver function test results, and the presence of the KF ring. After several months of DPA treatment, the patient developed typical symptoms of MG, including diplopia and bilateral fatigue drops (apokamnosis), which varied throughout the study. The result of the edrophonium test was positive and high levels of antibodies against the acetylcholine receptor (AChR-Abs) and muscle-specific kinase Abs (MuSKAbs) were found in the serum. As in the case of the patient with LE, after discontinuing DPA, zinc sulfate and additional pyridostigmine were started. MG symptoms disappeared and the number of autoantibodies gradually decreased [177].

All patients described in other reports [178,179,180,181,182,183] were teenagers, the majority of whom were women (75%); the duration of DPA treatment until the onset of MG symptoms was 2–12 months (but may be visible years after the start of treatment); symptoms were generally ocular. In three of them, DPA was discontinued and switched to another anti-copper drug, and symptomatic treatment of MG was implemented. All of them achieved an improvement in their neurological condition. Complete disappearance of side effects occurred within 2–7 months. DPA treatment was continued in two patients; in one, antimyasthenic therapy was started and significant improvement was observed; in the second case, MG was finally diagnosed, and DPA was probably the trigger for the onset of MG. Since myasthenia gravis is an autoimmune disease, immune-mediated effects of D-penicillamine may be observed, such as antibodies against the nicotinic acetylcholine receptor, thymic hyperplasia, abnormal T cells, and even antibodies against muscle-specific kinase [160].

**Figure 2:**
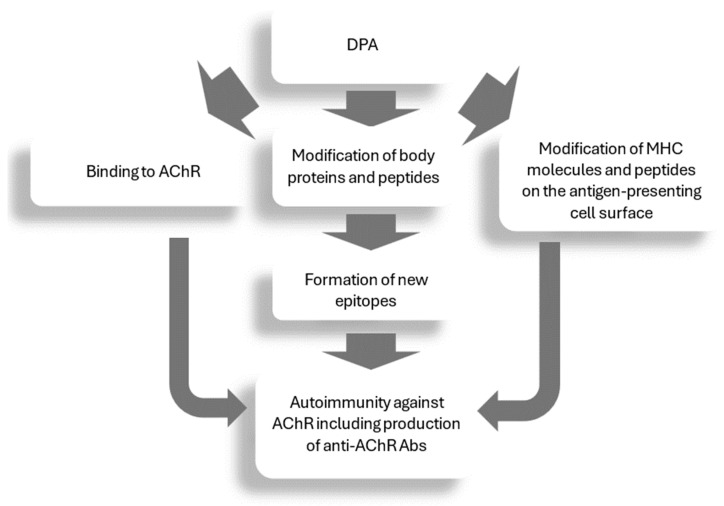
Potential immune mechanism of DPA-induced myasthenia gravis. DPA, d-penicillamine; Abs, antibodies; AChR, acetylcholine receptor; MHC, major histocompatibility complex.

The pathogenesis of DPA-induced MG is unclear, and the exact mechanism remains unknown. The increase in AChR-Abs, typical of idiopathic MG, may indicate a common pathogenesis. In the case of DPA-related MG, this may be due to DPA binding to AChR and altering the autoimmunogenicity of this complex, resulting in the production of AChR-Abs [184]. It has also been hypothesized that DPA may cause modifications in histocompatibility complex molecules and peptides on the surface of antigen-presenting cells, leading to autoimmunity against AChRs [185]. It also seems possible that, due to its molecular size and reactive sulfhydryl group, DPA may modify the body’s proteins or peptides, leading to the creation of new epitopes [185] (see Figure 2).

The presence of both AChR-Abs and MuSK-Abs suggests a common autoimmune reaction after DPA [185,186,187,188], as both are rare in idiopathic MG. Another explanation for this phenomenon is that DPA stimulates prostaglandin E1 (PGE1) synthetase, producing prostaglandin E1, which resides in the allosteric site of the AChR and inhibits acetylcholine binding [186,189].

**Figure 3:**
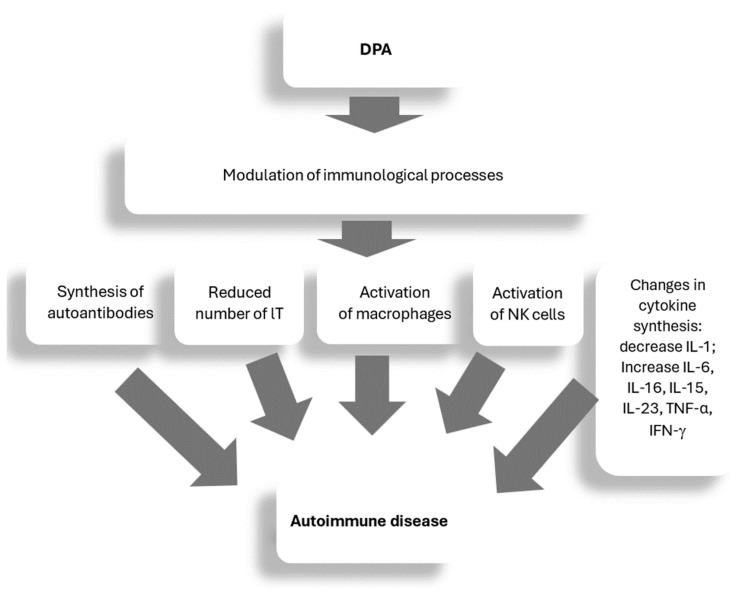
Potential mechanisms of DPA-induced autoimmunity. DPA, d-penicillamine; IFN-γ, interferon gamma; IL-1, interleukin-1; lT, T lymphocytes; NK cells, natural killer cells, TNF-α, tumor necrosis factor alpha.

DPA treatment has been shown to be associated with an increased risk of autoimmune diseases other than MG or lupus, such as glomerulonephritis, Goodpasture syndrome, vasculitis, polymyositis, ulcerative colitis, multiple sclerosis, polyarthritis, psoriasis, serpiginosa perforans, pemphigus, pemphigoid, and epidermolysis bullosa [65]. According to research by Seessle et al., 2.6% of the analyzed patients with WD may develop an autoimmune disease during DPA treatment [170]. The exact mechanism of the association between DPA autoimmunity in WD is unknown [190,191]. It has been shown that DPA can modulate immunological processes, including reducing the number of T lymphocytes and initiating the synthesis of autoantibodies and causing the activation of macrophage functions. In addition, it can lead to a decrease in serum levels of IL-1 and rheumatoid factor (RF), an increase in serum levels of IL-6, IL-13, IL-15, IL-23, TNF-α, and IFN-γ, and the activation of NK cells, which can often lead to autoimmune diseases and autoimmune complications of DPA treatment (see Figure 3) [9,170,190,191,192].

Seesle et al. conducted the first cohort study of 235 patients with WD to assess the incidence of concomitant or therapy-induced immunological diseases and to assess the role of ANA in therapy monitoring [170].

In the study cohort, 8.1% of patients had concomitant autoimmune diseases, of which 5.5% had a previous autoimmune disease and 2.6% developed an autoimmune disease after long-term treatment with DPA. The incidence of concomitant autoimmune diseases at the time of initial diagnosis of WD (14/235) was similar to the incidence of autoimmune diseases in the general population. Interestingly, the results of this study partially suggested a higher incidence of autoimmune diseases in patients with cirrhosis. This is surprising because cirrhosis is thought to be an immunosuppressive disease. Czlonkowska et al. described, in 1976, immune disorders in WD caused by liver cirrhosis and copper metabolism abnormalities [8]. They noticed increased levels of IgG and IgM and a reduced percentage of T lymphocytes, as well as reduced bactericidal activity in WD and in the group with liver cirrhosis compared to the control group. The changes were more pronounced in patients with WD than in patients with cirrhosis unrelated to WD. The authors speculated that free copper could have influenced lymphocyte functions [8].

Another interesting conclusion from the study by Seesle et al. [170] concerns the diagnosis of autoimmune diseases in 6/235 patients (see Table 3); all of these patients were treated with DPA. Except for one patient, these autoimmune diseases appeared after treatment with DPA (from 11 to 25 years).

The patients were diagnosed with ulcerative colitis, SLE, multiple sclerosis (MS), Werlhof’s disease, seronegative polyarthritis, and psoriatic arthritis. It is difficult to determine whether there was a causal relationship between DPA treatment and the development of autoimmune disease due to the large time interval between the initiation of treatment and the diagnosis of autoimmune disease. However, no autoimmune diseases occurred in patients treated with trientine or zinc. The authors therefore suggest that monitoring for possible autoimmune complications is necessary in patients treated with DPA [170].

Some autoimmune diseases are associated with the occurrence of characteristic antibodies. Their determination could serve as a screening test in monitoring the occurrence of undesirable autoimmune reactions during DPA treatment. However, the value of the ANA test is controversial because these antibodies are also detected in healthy people.

Seesle et al. reported no association between ANA titer and the development of autoimmune adverse events during DPA treatment [170].

It is worth mentioning that in addition to complications related to the autoimmune process, the use of DPA is associated with other serious immunological/inflammatory side effects. These include nephrotic syndrome, renal vasculitis, and bone marrow aplasia. Their prevalence is low. Mechanisms of DPA-induced renal injury may include direct toxicity or immunological and/or circulatory effects, which may involve various renal structures, such as glomeruli, tubules, interstitium, and vasculature [160].

### 5.2. Autoimmune Autoantibodies Not Related to Treatment of WD

Since numerous liver diseases, including autoimmune hepatitis, are associated with an increase in the production of autoantibodies, it can be assumed that not only DPA treatment, but also liver damage may lead to the appearance of autoantibodies in patients with WD and that immunological diseases/autoimmune processes may have an influence on the course of the disease. As mentioned earlier, in WD, advanced liver cirrhosis may lead to the development of immunological changes, immunodeficiencies, systemic inflammatory response, activation of circulating immune cells, and changes in cytokine concentrations. It cannot be ruled out that autoimmune processes may affect not only the course of liver damage but also the CNS, as previous studies have shown the association of antineuronal antibodies with various CNS pathologies.

The study by Antczak-Kowalska et al. showed a higher incidence of ANAs, ANCAs, neuronal surface autoantibodies (NSAbs), and onconuronal antibodies (ONAs) in patients with WD compared to the control group; at the same time, there was no difference in the occurrence of antibodies depending on the form of the disease (hepatic vs. neurological) and the method of treatment (DPA vs. zinc sulfate) [192].

The cause and clinical significance of ANA and ANCA antibodies in WD are unclear but may reflect immune system dysregulation (or may be an incidental observation). The same applies to NSAbs. Although previous studies have described the occurrence of these antibodies in patients with various neurodegenerative processes [193,194], in Antczak-Kowalska’s study there were no differences in the frequency of these antibodies between patients with hepatic and neurological forms of WD; treatment had no effect on the occurrence of antibodies, as there were no differences in their frequency between the groups of patients treated with DPA and zinc sulfate. Also, the detected presence of ONA in patients with WD is not easy to explain. The occurrence of classic ONAs is most often associated with cancer [195] and indicates the diagnosis of a paraneoplastic neurological syndrome. In the Antczak-Kowalska study, five patients had a positive history of cancer, but except for one case, they had no detectable ONA.

In the pediatric population, the presence of autoantibodies was significantly more common among WD patients than in the control group (84% vs. 37%, respectively) [196]. This was especially true for ANAs; the difference in the occurrence of ANCAs was not statistically significant, although smooth muscle antibodies (SMAs) and anti-parietal cell antibodies (APCAs) were detected more often in children with BD than in the control group. One patient had antibodies against tissue transglutaminase 2 (tTG2) IgA and antibodies against deamidated gliadin peptide (DPG); he was diagnosed with celiac disease. A positive ANA result was not associated with liver function and copper metabolism parameters or with transient elastography results. The type of treatment was not associated with the presence of autoantibodies. The authors emphasized that none of the patients with ANAs were ultimately diagnosed with an autoimmune disease. Therefore, based on these observations, a hypothesis regarding a higher incidence of autoimmune diseases in WD cannot be established. Some authors suggest that autoantibodies in WD may potentially be induced by hepatocyte necrosis, especially in the early stages of this disease [197].

Therefore, the importance of autoantibodies in WD remains unclear. However, the authors presented an interesting hypothesis, according to which the variability of clinical symptoms of WD related to the occurrence of hepatic/neurological symptoms at different ages may be related not only to the modifying influence of genetic and environmental factors but also to immunological ones, factors responsible for triggering the inflammatory process contributing to liver/CNS damage [198,199].

It is worth mentioning that WD may be associated with an autoimmune disease, regardless of DPA treatment. Some patients have been described in whom SLE coexists with WD [200,201,202,203,204,205,206,207,208,209] (see Table 4). Most of these patients were women; most of them were diagnosed with SLE simultaneously with WD, and most of them had hepatic disease. SLE is characterized by a complex and heterogeneous clinical picture that may involve several organs and tissues. An important target organ in SLE is the liver; according to published data, between 25% and 50% of SLE patients may have liver dysfunction [209,210]. It has been documented that SLE often causes a subclinical liver dysfunction called lupus hepatitis [211]. It is a nonspecific reactive liver disease mainly caused by complement accumulation and liver damage caused by vasculitis [212,213]. Perhaps it can be hypothesized that SLE-related liver damage may be a trigger for the clinical symptoms of WD. And even if this is not the case and, in the described patients, liver damage develops independently because of WD and SLE, special attention should be paid to WD in the differential diagnosis of patients with suspected SLE. When diagnosing WD, we must also remember that various symptoms, including liver symptoms, may have different etiologies at the same time. Particular attention should be paid to women because most autoimmune diseases are more common in females. Mageed et al. [208] prepared a useful table in which they described the features that differentiate the symptoms of WD and SLE. This information, with minor modifications, is presented in Table 5.

### 5.3. WD and Autoimmune Diseases

The hepatic form of WD must be differentiated from autoimmune hepatitis (AIH). Both diseases should be considered when investigating chronic liver disease with negative viral serology [214,215].

AIH is a chronic liver disease characterized by humoral and cellular reactions against antigens, mostly against host hepatocytes. The typical laboratory results of patients with AIH are characterized by an elevation of aminotransferases, hypergammaglobulinemia, or elevation of IgG and significant titers of selected autoantibodies, including antinuclear antibodies, anti-liver–kidney microsome type 1 antibodies, smooth muscle antibodies, and soluble liver antigen/liver–pancreas antibodies. Extrahepatic manifestations of AIH are reported in one-fourth of patients [216]. They frequently overlap with other liver autoimmune diseases, like primary biliary cholangitis, or primary sclerosing cholangitis, observed in patients with AIH. AIH used to be termed “lupoid hepatitis” because of the presence of ANAs; however, most patients with AIH do not present SLE.

Several cases of WD were described that were initially diagnosed as AIH; partial response to steroids and azathioprine was achieved in them. For that reason, patients initially diagnosed as AIH should be screened for WD, especially if they poorly respond to immunosuppression [211]. Both the American Association for the Study of Liver Diseases (AASLD) and the European Association for the Study of the Liver (EASL) include WD as a differential diagnosis to AIH [214,217,218].

There are several biochemical and histopathological features that enable differentiation between WD and AIH.

One of the useful diagnostic methods differentiating WD and AIH is liver biopsy and histochemical staining. Histochemical stains for copper or copper-related proteins, such as rhodamine, provide qualitative evidence of increased hepatic copper [197,219].

Despite elevated liver copper, these stains are often negative in patients with WD. Another test that confirms WD in patients is the 24 h urine copper. This test is abnormal in 80–85% of untreated WD patients. However, in any case of severe icteric hepatitis, there may be disorders of copper metabolism. Although the 24 h urinary copper concentration is sometimes elevated in acute icteric hepatitis, this level does not exceed 200 micrograms/24 h.

Low ceruloplasmin levels are seen in most patients with neurologic WD but may be within normal range in about half of the patients with Wilson’s liver disease [220], while the presence of KF rings is observed in about 50% of cases at diagnosis [221]. The elevation of urinary copper is also an important marker for diagnosis, and liver biopsy is recommended when the etiology is still uncertain.

In AIH, symptoms such as fatigue, malaise, and rashes may occur; laboratory test results and detection of increased levels of antibodies, including total IgG, ANAs, and ASMAs, are important. However, it has to be remembered that, especially in the early stages of this disease, hepatocyte necrosis and intracellular antigen exposure to the immune system may result in low titer autoantibody production in WD patients, and it is a misleading point in differentiating AIH from WD [222]. However, in cases with mild symptomatology and in the setting of fulminant hepatic failure, proper diagnosis is a real challenge. In the latter case, frequently, a correct diagnosis is established after histopathological examination of the explanted liver [223].

A few cases of overlap of WD and AIH have been described (see: Table 6). But available cases and data collected do not suggest a predisposition of WD patients to develop AIH. More frequently, patients with WD are misdiagnosed with AIH and vice versa.

In medical literature there are available a few reports of AIH coexisting with WD. They have been excellently reviewed by Nasri et al. [224]. All of them described children or young adults [197,215,222,224].

As the treatments for AIH and WD are substantially different, AIH must be always included in the differential diagnosis of WD regardless of the stage of the disease [214]. This is of particular importance in cases of AIH or WD not responding to immunosuppression or anti-copper treatment, respectively [89].

Additionally, it is worth mentioning that, apart from the autoimmune mechanism involved in liver damage in WD (described above), there are several case reports describing co-occurrence of the neurological autoimmune disorder multiple sclerosis (MS) in WD patients [225,226,227].

As MS is an autoimmune demyelinating disease affecting the CNS with unknown etiology, the possible significance of environmental factors (e.g., involvement of metals and metabolism disturbances, including copper and zinc) [228,229] are hypothesized in MS etiology. However, contrary to WD, the copper deficiency may lead to CNS demyelination and symptoms like those of MS [229]; further, WD patients with MS described in the literature had a mostly benign course of MS. The direct immunosuppressive effect of high “free” copper levels on T-cells, as well as DPA, may act additionally as an immunosuppressive drug (used in rheumatoid arthritis—the mode of action described above). However, further studies, especially in MS patients according to copper significance, are needed to verify these interesting findings.

**Table 6:** Clinical characteristics of patients with WD and AIH coexistence.

Age, Sex	Clinical Characteristics	Reference
15 years old, F	Acute hepatitis, Wilson disease diagnosed, no satisfying response to anti-copper drugs; ANAs 1:40, ASMAs 1:40, IgG 15.4 g/L, glucosteroids added to treatment regimen with good response	[230]
24 years old, F	Chronic hepatitis, ASMAs 1:60, ANAs 1:40, IgG 24.6 g/L. Liver biopsy: cirrhosis with moderate, periseptal inflammatory activity (interface hepatitis). Deposits of copper in the periseptal regions. Deteriorated on steroids within 3 years, treated with liver transplantation, copper content in explanted liver 965 mg/g	[215]
22 years old, F	Acute liver failure, ANAs 1:400, ASMA-positive, ceruloplasmin 11.2 mg/L, urine copper excretion 187.5 µg/21 (normal range 10–60 µg/24 h), Kayer–Fleischer ring present.; the patient died	[222]
30 years old, F	Chronic hepatitis, IgG elevated, ANA-positive, liver biopsy piecemeal necrosis, interface hepatitis, AIH diagnosed, on steroids 3 years with improvement, thereafter neurological symptoms appeared. The serum ceruloplasmin was decreased normal 24 h urinary copper excretion. Kayser–Fleischer present. *ATP7B* mutation confirmed. Head MRI showed the hyperintensity in bilateral basal ganglia. DPA was introduced with improvement.	[231]
10 years old, M	Acute hepatitis, ANAs 1:160, AMAs 1:160, ASMAs 1:80, Anti-LKM1 1:20, Kayser–Fleischer ring present, liver biopsy interface hepatitis, copper in dry liver tissue, 20 times the upper limit, steroids and DPA introduced, full recovery	[197]
15 years old, F	Acute hepatitis, ANA-, ASMA-, and AMA-positive, IgG 2870mg/dL, *ATP7B* c.2532delA and c.3061-1 G->A, Serum ceruloplasmin: 13.7 mg/dL, Liver copper content: 388 mg Cu/g dry tissue. Failed on steroids and DPA, finally treated with liver transplantation	[232]

ANAs, anti-nuclear antibodies; ASMAs, anti-smooth muscle antibodies; AIH, autoimmune hepatitis; Cu, copper; DPA, d-penicillamine; F, female; LKM1, liver kidney microsome type 1; M, male.

### 5.4. Possible Patomechanisms of Autoimmunity in WD

The pathogenesis of autoimmune processes in WD is not fully explained. As for the pathomechanism of autoimmune processes that are complications of treatment, it was characterized in earlier chapters. As for the pathomechanism of autoimmune diseases unrelated to treatment that reduces excess copper in the body, it has not been established yet. Therefore, when considering the cause of the occurrence of autoantibodies/autoimmune diseases in patients with WD, one must only rely on general knowledge regarding immunological/autoimmune processes. 

Autoimmune processes are believed to be complex and result from the interaction of genetic and environmental factors. Variants in various genes can cause defective regulation or a lowered activation threshold of lymphocytes, and environmental factors initiate or increase the activation of self-reactive lymphocytes that are out of control and ready to react against self-antigens. Common environmental factors that are involved in the development of autoimmune diseases include bacteria, viruses, and xenobiotics, such as chemicals, drugs, and metals [233].

The development of an autoimmune reaction may be caused by molecular mimicry (similarity of antigenic structures/epitopes present on pathogen cells and the body’s own cells), disturbances in the regulation of the response to own antigens (related e.g., to the dominance of Th1 or Th2 cytokines), polyclonal activation (some of them are capable of this, such as microorganisms that can cause activation of specific immune cells regardless of the TCR receptor), modification of autoantigens by microorganisms, metals, or drugs (this may be related to the absorption of foreign molecules on the surface of body cells or a reaction of foreign molecules with cell surface antigens affecting the change in immunogenicity), abolition of anatomical/molecular sequestration of self-antigens, disorders of immunological regulation (disorders of idiotypic regulation or disorders related to T or B lymphocyte anergy) [234].

The mechanisms responsible for the occurrence of autoantibodies in patients with WD are not known. As mentioned earlier, autoantibodies in WD are not related to the treatment or the form of the disease. The importance of these antibodies and their potential impact on the course of the disease are also unclear. So, it may be hypothesized, that variability of clinical symptoms of WD, related to the occurrence of hepatic/neurological symptoms at different ages, may be related not only to the modifying influence of genetic and environmental factors, but also to immunological factors responsible for triggering the inflammatory process contributing to liver/CNS damage [198,199]. But this hypothesis has not yet been verified in research.

Some authors suggest that autoantibodies in WD may potentially be induced by hepatocyte necrosis, especially in the early stages of this disease [197].

It seems possible that the autoimmune reaction in WD may be related to the chronic inflammation and tissue damage caused by copper toxicity, as discussed in earlier chapters. Recently, increasing evidence indicates that an abnormal inflammatory response is closely associated with many autoimmune diseases, including rheumatoid arthritis (RA), inflammatory bowel disease (IBD), SLE, and diabetes [235,236,237,238]. Although the importance of inflammatory dysregulation in chronic diseases has been reported in recent studies, the pathogenesis of inflammatory dysfunction in autoimmune diseases remains elusive.

Cell and tissue damage associated with inflammation and, in the case of copper, also with oxidative stress, generates a number of immune-enhancing signals, including ROS and DAMP, that stimulate sentinel cells such as dendritic cells (DCs), macrophages, and monocytes, for antigen uptake and activation and then, migration to regional lymph nodes, where the antigen is presented to T and B lymphocytes. After activation, these cells reach cells and tissues expressing the autoantigen, which may lead to the intensification of the autoimmune process.

Recent compelling evidence has shown that abnormal T cell immune response, including Th1, Th2, and Th17 cell responses, was actually having a crucial role in the inflammation of autoimmune diseases [239]. In addition to T cells, many other immune cells are implied in the development of autoimmunity. B cells are best known for their capacity to produce antibodies, which often play a pathogenic role in autoimmune diseases [240,241].

#### Can Autoimmune Processes in WD Be Directly Caused by Copper?

Clinical experience and the scientific literature have established that metals may play an important role in the development of autoimmune diseases. However, no data on the effects of copper on auto-reactivity has been published so far, except for the works mentioned above, which did not investigate the mechanisms responsible for the production of autoantibodies/the occurrence of autoimmune disease. Therefore, one can only put forward hypotheses that require verification in future research.

Metals in ionic form are quickly bound to blood proteins, the endothelium, and blood cell membranes, especially to the water-soluble components of lipoproteins [242]. Copper has been shown to have a high affinity for molecules containing sulphydryl (SH) groups and thiol groups, such as methionine, cysteine, and glutathione [243]. Copper bound to thiols and other groups can cause protein modifications and the formation of altered self-protein with new antigens, which, previously unknown to the immune system, will become the target of an autoimmune reaction, activating B cells with the participation of Th lymphocytes [233,244,245]. Due to cross-reactions, T cells may later also react with the native unaltered self proteins.

Additionally, copper, after binding to molecules/structures containing SH groups, may contribute to the generation of free radicals that will modify autoantigens, revealing epitopes that may trigger autoimmunity.

Binding of metals directly to major histocompatibility complex II (MHC II) without prior processing by antigen-presenting cells or even directly to the T cell receptor has also been suggested as a mechanism of metal actions associated with autoimmunity [246]. There is further evidence that metals can cause aberrant MHC II expression on target cells, inhibit T suppressor cells, cause changes in the idiotype–anti-idiotype network, and induce heat shock proteins [247,248]. These and other factors may play a role in metal-induced autoimmunity. Such mechanisms have never been studied for copper.

In case of copper, its role in autoimmunity may also be related to a possible immunomodulatory effect resulting from its influence on the production of cytokines and activation of Th1 and Th2 cells. Incorrect activation of these cells may result in immune dysregulation leading to an impaired cellular and/or humoral response, which may lead to an autoimmune reaction [249,250].

A certain role in mediating the relationship between copper and autoimmunity may be played by the previously mentioned HMGB1 protein. It has been shown that, if located in an extracellular location, it can promote the pathogenesis of inflammatory and autoimmune diseases. The role of this protein depends on the redox state of its three cysteines at positions 23, 45, and 106, which are critical for activity. Fully oxidized HMGB1 is inactive; the partially oxidized HMGB1 can induce cytokine production via TLR4 or promote chemotaxis by binding the chemokine CXCL12 for stimulation by CXCR4 [251,252,253]. The role of HMGB1 in acute inflammation was defined, but its intriguing immunological properties have led to research investigating its role in chronic inflammatory and autoimmune processes. As for the latter, so far, its role in the pathogenesis of rheumatoid arthritis has been best studied, in which increased levels of HMGB1 in body fluids, increased expression in the affected tissue, and the ability to induce tissue pathology (e.g., inflammation) have been observed.

HMGB1 can induce cytokine synthesis, stimulate chemotaxis, activate cells, and can also be an adjuvant stimulating the production of autoantibodies [251]. HMGB1 acts by binding to various receptors, including RAGE and TLR2, TLR4, and TLR9 [254,255]. Binding to TLR4 may contribute to the activation of the NF-κB transcription factor, which increases the production of cytokines, including TNF-α and IL-6, by macrophages. HMGB1 may also bind to pathogen-associated molecular patterns (PAMPs), lipopolysaccharide (LPS), RNA, DNA, cytokines, or receptors for cytokines and induce the production of various mediators of the inflammatory reaction [256].

Why does the presence of autoantibodies most often not lead to the development of autoimmune disease in patients with WD? It is difficult to explain, but perhaps it is related to immunosuppression, as described in the 1970s, the mechanism of which was explained in earlier chapters.

**Figure 4:**
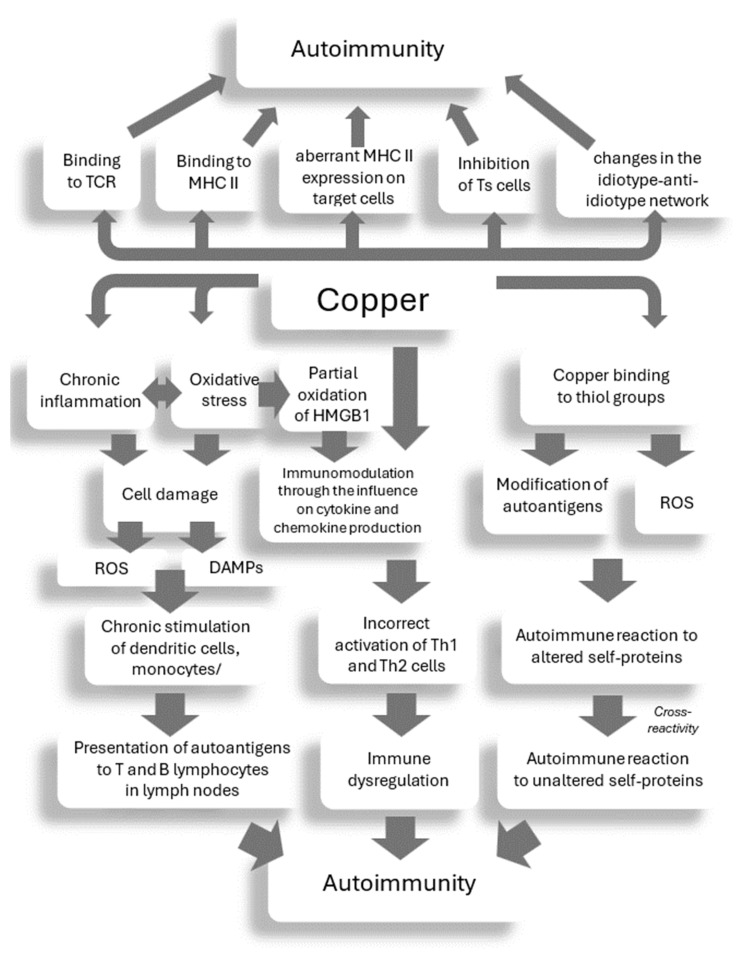
Possible mechanisms linking copper to autoimmunity. DAMP, damage-associated molecular pattern; HMGB1, high-mobility group box 1; ROS, reactive oxygen species.

Possible mechanisms linking copper to autoimmunity are presented in Figure 4.

### 5.5. Steroids in WD Treatment

Since autoimmune reactions have been observed during WD treatment, the advisability of using anti-inflammatory drugs (steroids or immunomodulation) in this disease was considered [215,257,258,259,260]. There are several situations in which such treatment should be considered in clinical practice, particularly in the setting of hepatic WD.

The first situation concerns the coexistence of WD and AIH, an autoimmune and necro-inflammatory liver disease of unknown origin [215,257,258,260]. AIH is characterized by the presence of autoantibodies (ANAs, anti-smooth muscle actin (SMA) antibodies, anti-liver kidney microsomal (LKM) antibodies), hypergammaglobulinemia, increased activity of liver enzymes (transaminases), and specific histological features, with a positive response to steroid treatment.

There are many cases in the literature of patients diagnosed first as AIH, then as WD or variant WD, and finally, as AIH or a coincidence of WD and AIH. Differential diagnosis of both diseases in case of liver injury is necessary. Treatment with oral steroids, even in patients with WD initially diagnosed as AIH, gave very good results [215,257,258,260]. Such treatment is also necessary for patients suffering from two diseases at the same time.

The second situation occurs, as mentioned in the previous sections, during treatment with anti-copper (especially DPA) in patients with WD, who may develop autoimmune diseases and direct toxic effects. Treatment of such events usually involves discontinuation of DPA (and the use of another anti-copper drug), but in more severe cases, temporary treatment with steroids is suggested to reduce the autoimmune response and clinical symptoms associated with a DPA-related adverse event [160]. In some cases, such as DIL or MG caused by DPA, such treatment is even recommended if the diagnosis is confirmed until symptoms disappear [160].

Another possibility of using steroids in the treatment of an adverse drug reaction caused by DPA is the so-called steroid desensitization; starting premedication with oral prednisolone 2 days before reintroducing DPA at a reduced dose leads to better tolerance of DPA and avoidance of autoimmune reactions associated with DPA [261].

Additionally, based on theoretical data on inflammation in neurological WD, it can be hypothesized that immunosuppression may attenuate astroglial and microglial responses that lead to neuronal damage, but such a hypothesis must first be tested in animal studies [262].

## 6. Limitations, Summary, Conclusions and Future Perspectives

This study has potential limitations. Firstly, the mechanism of the involvement of pro-inflammatory/immune processes in the pathogenesis of tissue damage in WD is still poorly understood. Little research has been done on this issue because WD is a rare disease and it is difficult to obtain material suitable for research, such as liver tissue, brain tissue, or cerebrospinal fluid. Therefore, knowledge about inflammatory/immune mechanisms in WD comes from experimental studies using animal models of this disease or is based on the measurement of inflammatory markers in the blood, which do not necessarily reflect processes occurring in tissues.

Conclusions about the main pathomechanisms in the pathogenesis of CNS damage in WD based on the results of tests of inflammatory/immune parameters of blood may be misleading because individual components may be of hepatic, not brain origin. However, it is worth mentioning that the relationship of specific parameters with the hepatic or neuropsychiatric form of the disease may indicate their role in the pathogenesis of liver or CNS damage.

Another limitation is that we report data on patients diagnosed in different countries that use different diagnostic criteria for WD. In Europe, the diagnostic scheme developed in Leipzig or the subsequently modified Leipzig scoring method of 2019 are most commonly used [263,264], but different criteria and methodologies are used in other parts of the world. Therefore, there is a small risk that the patients mentioned were not correctly diagnosed. Although it is important to remember that information about patients comes from publications reviewed by reviewers who are researchers with extensive experience in a given topic. Therefore, it can be assumed that even if the Leipzig criteria were not used to diagnose WD, the diagnosis of WD was made correctly.

It should also be noted that most information about autoimmune processes in WD is based on case observations. Case reports are descriptive and not based on evidence; this is the nature of this type of work. WD is a rare disease, and it is difficult to conduct large clinical trials. Much information about this disease can be obtained from observing individual cases.

Another limitation of this work is that we could not access all published case reports, especially those in French, Slovak, and Spanish.

However, our work is the first to provide comprehensive information on inflammatory/immune/autoimmune processes in WD, including molecular mechanisms, based on knowledge about WD or resulting from research on the described processes and phenomena.

Future research should focus on determining the importance of autoimmune processes in the pathogenesis of side effects of drugs used in WD, especially DPA, determining the usefulness of the use of corticosteroids in the prevention of autoimmune problems, and determining when and in what doses these drugs should be used. Biomarkers useful in identifying patients for such treatment should also be identified. The clinical significance of the many different autoantibodies present in patients with WD also remains to be determined.

A better understanding of the molecular mechanisms of inflammatory/ immune/ autoimmune processes may be important for better understanding the causes of the diversity of symptoms, varied clinical course, and response to therapy, as well as for the development of new treatment regimens for WD.

## Figures and Tables

**Table 1:** WD patients in whom SLE post D-penicillamine (DPA) treatment was diagnosed.

Patient Description Sex, Age at WD Diagnosis	WD Form	Age at AID Diagnosis	Time of Treatment before AID Onset (Years)	AID	ANA	Treatment Decision after SLE Diagnosis	Did SLE Symptoms Disappear after Discontinuing DPA?	Reference
Female 22	P	23 y	1 year	SLE symptoms (polyarthralgia, pericarditis, pleurisy, fever) and aortic regurgitation	ANA (1/500) and antinucleoprotein antibodies	Discontinuation DPA, corticosteroids	Yes, after 6 months	[166]
Sex * 8	*	*	*	SLE	“serologic changes of SLE”	In 4 discontinuation of DPA treatment	*	[167]
Female 19	P	22 y	3 y	arthritis	ANA of 1:640 (homogeneous), C3 35 mg/dl and anti-DNA 85% lupus anticoagulant was detected, anti-DNA of 1:2560	tetraethylene tetramine dihydrochloride was substituted for DPA, steroids; liver transplantaion	Yes (after liver transplantation)	[168]
Female 5	?	7 y	2 y	SLE	arthritis, malar rash and laboratory findings suggestive for lupus erythematosus	zinc acetate was substituted for penicillamine	*	[169]
Male 9	M	22 y	11 y	SLE seronegative polyarthritis	elevated ANA titers (>1:160)	Zinc	*	[170]
Male 23	NP		1 y	SLE fever with arthralgia of the shoulders, hips, knees, feet, and thoracic spine	lupus anticoagulant and antinuclear antibodies (ANAs); anti-dsDNA, c-and p-ANCA, CCP antibodies (-)	Zinc sulphate, steroids	Yes, over 7 weeks	[176]

AID, autoimmune disease; P, presymptomatic; NP, neuropsychiatric; M, mixed; SLE, systemic lupus erythematosus; ANAs, anti-nuclear antibodies. * The lack of data.

**Table 2:** WD patients in whom myasthenic syndrome post D-penicillamine (DPA) treatment was diagnosed.

Patient Description Sex, Age at WD Diagnosis	WD Manifestation	Age at AID Diagnosis	Time of Treatment before AID Onset (Years)	ANA	Treatment Decision after SLE Diagnosis	Did SLE Symptoms Disappear after Discontinuing DPA?	Reference
Male 56 y	H	58 y	15 months	AChR-Abs (+) MuSKAbs (+)	Withdrawal of DPA zinc sulphate pyridostigmine	At the follow-up visit after 6 months the patient did not present MG symptoms	[177]
Male 9 y	NP	15 y	6 years	AChR-Abs (+) MuSK-Abs (-)	Withdrawal of DPA Pyridostigmine	Remarkable improvement within 2 months	[178]
Female 11 y	H	17 y	6 years	AChR-Abs (+) MuSK-Abs not tested	DPA Pyridostigmine	Remarkable improvement	[179]
Female 15 y	P	16 y	4 months	AChR-Abs and MuSK-Abs not tested	Trientine instead of DPA clobetasone butyrate for skin lesions (indication dermopathy)	One month later disappearance of the visual and skin complaints At follow-up at 7 months, complete resolution of all DPA-associated side effects	[180]
Female 8 y	H	12 y	4 years	AChR-Abs (+) MuSK-Abs not tested	Withdrawal of DPA trientine	Remarkable improvement within 3 months	[181]
Female 10 y	H	18	8 years	AChR-Abs (+) MuSK-Abs not tested	DPA pyridostigmine and neostigmine	Increased serum AChR-Abs were observed during follow-up	[182]
Male 13 y	P	14 y	13 months	AChR-Abs (+) MuSK-Abs not tested	Withdrawal of DPA Neostigmine	Remarkable improvement with cessation of all symptoms within 6 weeks	[183]

AID, autoimmune disease; Abs, antibodies; AChR, acetylcholine receptor; ANAs, anti-nuclear antibodies; DPA, d-penicillamine; NP, neuropsychiatric; M, mixed; MuSK, muscle-specific kinase; P, presymptomatic.

**Table 3:** WD patients in whom different AID post D-penicillamine (DPA) treatment were diagnosed (described in [170]).

Patient Description Sex, Age at WD Diagnosis	WD Form	Age at AID Diagnosis	Time of Treatment before AID Onset (Years)	AID	ANA Elevation	WD Treatment Decision
Female 27	N	38 y	11 y	Ulcerative colitis	At diagnosis (−) Following second-line therapy (+)	Zinc
Male 23	M	24 y	1 y	SLE	At diagnosis (−) Following second-line therapy (+)	Zinc
Female 17	H	39 y	22 y	MS	At diagnosis (−) Following second-line therapy (−)	Zinc
Female 16	H	28 y	12 y	Morbus Werlhof	At diagnosis (−) Following second-line therapy (−)	Trientine
Male 9	H	20 y	11 y	Polyarthritis (seronegative)	At diagnosis (−) Following second-line therapy (+)	Zinc + trientine
Female 8	P	32 y	24 y	Psoriasis with psoriatic arthritis	At diagnosis (−) Following second-line therapy (+)	Trientine

AID, autoimmune disease; ANAs, anti-nuclear antibodies; H, hepatic; MS, multiple sclerosis; M, mixed; N, neuropsychiatric; SLE, systemic lupus erythematosus.

**Table 4:** WD patients in whom different WD and SLE not related to DPA treatment were diagnosed (described in [200]).

Patient’s Sex	WD Manifestation	Age at WD/AID Diagnosis	Disease Sequence	Main Symptoms	Immune/Autoimmune Laboratory Tests Results	Reference
Female	H	18/18	SLE/WD simultaneous	rashes on face and trunk together with icterus, pruritis, and fatigue; mild anemia and thrombocytopenia; elevated liver enzymes, hypoalbuminemia, and bilrubinemia; erythematous and slightly scaly macular papules on both cheeks and the neck and upper back, hepatomegaly, splenomegaly, and KF rings in both eyes. Decreased serum ceruloplasmin and copper, elevated urinary copper. Cirrhosis and splenomegaly	hypocomplementemia, ANA, anti-dsDNA antibodies, and anti-β2GP1 antibodies (+); Coombs test (−), bone marrow smear showed marked hyperplasia and defective megakaryocyte maturation consistent with immune thrombocytopenia.	[200]
Female	FH	12/10.5	WD after 1.5 years of SLE	nephritis, pleurisy, hemolytic anemia; fulminant hepatic failure; decreased serum ceruloplasmin level and the presence of KF ring	ANA, anti-dsDNA, Coombs test (+)	[201]
Female	H	12/12	SLE/WD simultaneous	abnormal LFT; abnormal copper metabolism; Kayser–Fleischer rings; nephritis	complement C3 decreased; ANA, anti-dsDNA, LA	[203]
Female	NP	18/18	simultaneous WD/SLE/secondary Sjögren syndrome with aPL antibodies.	abnormal limb movements and slurred speech; dysdipsia, unsteady gait, dyskinesia, significantly increased involuntary movements of limbs; KF ring in both eyes; nephritis, fever, oral ulceration, xerostomia, keratoconjunctivitis sicca; Sjögren syndrome	elevated ESR, C-reactive protein, IgG, and IgM, and hypocomplementemia; ANA, anti-SSA antibody, anti-rRNP antibodies; anti-ACL antbodies, LA and anti-β2GP1 antibodies (+)	[204]
Female	H	32/29	simultaneous	recurrent fever and multi-joint pain; liver cirrhosis, liver fibrosis, K–F ring	Leucopenia, anemia, multiple auto-antibodies, ANA anti-dsDNAanti-SSA anti-Smith antibody, anti-Ro52 antibody and anti-rRNP antibody, low levels of serum complement components (C3, C4).	[205]
Female	NP	?/24	SLE with antiphospholipid syndrome, next WD	fever, neuropsychiatric symptoms, abnormal copper metabolismsm, KF rings;	?	[202] *
Female	H	17/17	Simultaneous WD/ SLE with APLA syndrome	facial and limb edema, abdominal distension, ascites, and splenomegaly. Abnormal LFT, low platelets; liver cirrhosis with splenomegaly.	Increased IgG, IgM; positive tests for antibodies against Sjögren’s syndrome A antigen (SSA), lupus anticoagulant (LA), and APLAs, including anti-domain I *β*2GPI antibodies, anti-vimentin antibodies, and anti-annexin A2 antibodies. The anti-dsDNA (−). Decreased complement 3 and C4. ANA (+), LA (+); AIH-related autoantibodies, such as ASMAs and LKM1 antibodies (−)	[206]
Male	H	12	SLE Simultaneous	hepatic cytolysis, and hepato-cellular insufficiency; impaired liver function, hemolytic anaemia, and normal alkaline phosphatase levels; abnormal copper metabolism; nephrotic syndrome and the presence of inflammatory syndrome; the kidney biopsy histopathology revealed nephritis lupus class II	native anti-DNA and anti-PCNA antibodies (+)	[207]
Female	N/M	19/16	SLE then WD	slurred speech, mild difficulty with deglutition, and an abnormally hyperextended right big toe; symmetrical basal ganglia abnormal signal intensity; severe acute hemolytic anemia; arthralgia of both knees, oral ulcers, photosensitivity, and proteinuria; thrombotic microangiopathy joint pain; abnormal LFT,	Smooth muscle antibody (SMA) and liver kidney microsomal type 1 (anti-LKM-1) antibodies (−) ANA (+), anti-dsDNA equivocal, and APLA (−)	[208]
Female	H	9/9	Simultaneous	red-colored urine, thrombocytopenia, anemia, abnormal LFT, SLE and lupus nephritis (LN) Under light microscopy 2 focal segmental sclerosis with balloon adhesions and one case of cell-fibrous neoplasm formation in 26 glomeruli. Diffuse light-moderate proliferation was present in the mesangial zone. Podocyte swelling, swelling of epithelial cells, and focal fibrosis of the renal interstitium.	ANA (+), anti-dsDNA (+), A kidney biopsy was performed to determine the renal pathology. We detected IgA(+), IgG(+), C3(+), F+(−), IgM(++), and C1q(++)	[209]

* lack of complete data due to lack of access to the full-text version of this publication. AID, autoimmune disease; AIH, autoimmune hepatitis; ANAs, anti-nuclear antibodies; ACLs, anticardiolipin antibodies; APLAs, anti-phospholipid antibodies; ASMAs, anti-smooth muscle antibodies; β2GP1, β2-glycoprotein 1; dsDNA, double-stranded DNA; K-F ring, Kayser–Fleischer ring; LA, lupus anticoagulant; LKM-1, liver/kidney microsomal; P, presymptomatic; NP, neuropsychiatric; M, mixed; SLE, systemic lupus erythematosus; ANAs, anti-nuclear antibodies.

**Table 5:** Some clinical features/symptoms in WD and SLE.

WD	Feature	SLE
-	Photosensitivity and malar rash	+
+ (after DPA)	Oral ulcers	+
+ (after DPA)	Proteinuria	+
Rare, may be the initial manifestation	Hemolytic anemia	+
+ (extrapyramidal)	Neuropsychiatric symptoms (CNS involvement)	+ (lupus cerebritis, TTP)
Rare	Arthralgia	+
+ (pathognomic)	Kayser–Fleischer ring	-

CNS, central nervous system; DPA, d-penicillamine; TTP, thrombotic thrombocytopenic purpura (according to [208], modified).

## Data Availability

No new data were created or analyzed in this study.
